# Derivation and Pluripotency Validation of Six iPSC Lines From Amniotic Fluid Carrying Intermediate α‐Thalassemia Genotypes (‐‐^3.7^/α^SEA^ and ‐‐^4.2^/α^SEA^)

**DOI:** 10.1155/sci/9951684

**Published:** 2026-06-03

**Authors:** Qingyun Chen, Xinxing Jiang, Xinhuai Xue, Yao Zhou, Yuanhua Huang, Fei Sun, Yanlin Ma

**Affiliations:** ^1^ Key Laboratory of Reproductive Health Diseases Research and Translation of Ministry of Education, Department of Reproductive Medicine, The First Affiliated Hospital, Hainan Medical University, Haikou, China, hainmc.edu.cn; ^2^ Key Laboratory of Human Reproductive Medicine and Genetic Research of Hainan Province, The First Affiliated Hospital, Hainan Medical University, Haikou, China, hainmc.edu.cn; ^3^ Hainan Provincial Clinical Research Center for Thalassemia, The First Affiliated Hospital, Hainan Medical University, Haikou, China, hainmc.edu.cn

## Abstract

**Purpose:**

Thalassemia intermedia (TI), or hemoglobin H (Hb H) disease, presents significant clinical heterogeneity, and its pathogenesis remains unclear. This study aimed to establish induced pluripotent stem cells (iPSCs) from amniotic fluid (AF) cells of fetuses diagnosed with intermediate α‐thalassemia genotypes (‐α^3.7^/‐‐^SEA^ and ‐α^4.2^/‐‐^SEA^) to provide a robust in vitro model for investigating disease mechanisms and exploring therapeutic strategies.

**Methods:**

AF cells from six TI fetuses were reprogrammed using nonintegrating episomal vectors via electro‐transfection. The resulting iPSC lines underwent comprehensive characterization, including alkaline phosphatase (AP) staining, immunofluorescence (IF) for pluripotency markers (e.g., OCT‐4, SOX‐2, and TRA‐1‐60), quantitative reverse transcription real‐time PCR (RT‐qPCR) for the expression of pluripotency genes, G‐band karyotyping, and genetic analysis for thalassemia. Differentiation potential was assessed in vivo via teratoma formation in immunodeficient mice and in vitro through directed hematopoietic differentiation.

**Results:**

Six stable iPSC lines were successfully established. All lines retained the progenitor’s intermediate α‐thalassemia genotype and exhibited normal karyotypes. They displayed typical embryonic stem cell (ESC)‐like morphology, were positive for AP, and expressed key pluripotency markers. In vivo, all lines robustly formed teratomas containing tissues derived from all three germ layers (endoderm, mesoderm, and ectoderm). Furthermore, in vitro hematopoietic differentiation protocols successfully induced the iPSCs to generate CD34^+^/CD43^+^ hematopoietic progenitors, which were functionally validated by their ability to form hematopoietic colonies in colony‐forming unit (CFU) assays.

**Conclusion:**

We have successfully derived and comprehensively characterized six patient‐specific iPSC lines from intermediate α‐thalassemia fetuses. These cell lines demonstrate robust pluripotency and maintain the capacity for hematopoietic differentiation. They represent a valuable resource for investigating TI pathophysiology, screening novel drugs, and developing future cell‐based therapeutic strategies.

## 1. Background

Thalassemia is one of the most common monogenic disorders in humans. It is an autosomal recessive condition that results from a deletion or mutation of the globin gene. According to the pathophysiology and the type of globin gene deletion or dysfunction, the diseases can, therefore, be grouped into α‐Thalassemia, β‐Thalassemia, δ‐Thalassemia, γ‐Thalassemia, and the relatively rare δβ‐ Thalassemia [1].

There are three main characteristics of α‐Thalassemia: widespread distribution, significant heterogeneity, and high mortality of the severe form. This condition not only poses a significant threat to the health of the world’s population but also imposes a heavy economic burden on society and the patient’s families [2, 3]. Thalassemia intermedia (TI), also known as hemoglobin H disease (Hb H), is the most severe non‐fatal form of α‐thalassemia, characterized by a wide range of clinical manifestations. Hb H disease loses three α‐genes (‐‐/‐α or ‐‐/α^T^α) and reveals mild to severe microcytic, hypochromic, and hemolytic anemia. Hb H disease is characterized by anemia (2.6–13.3 g/dL) accompanied by varying amounts of Hb H (0.8%–40%) and occasionally by Hb Bart’s in the peripheral blood [2]. As they age, inefficient erythropoiesis leads to splenomegaly, hepatomegaly, and increased marrow hyperplasia, resulting in skeletal skewing and characteristic facial structure alterations [4, 5]. Hb H disease is often considered a less severe disease process. However, recent studies have shown that, especially in patients with non‐deficiency Hb H disease, the condition may be far more severe than previously thought. These patients have more severe anemia, pronounced splenomegaly, and even transfusion dependence [6, 7]. In addition, the complexity of genetic counseling is further increased because the molecular regulation of this phenotype heterogeneity is not fully understood. The high phenotypic heterogeneity in Hb H disease and regional differences in treatment strategies further complicate disease management [8].

Scientists have primarily edited specific genes in animal models to establish hematologic disease models and investigate the developmental patterns of hematopoietic differentiation under pathological conditions due to ethical and legal constraints. There are disparities in development, gene expression, and epigenetics between animals and humans, so using animal models to mimic human hematopoiesis and diseases is not entirely precise. Induced pluripotent stem cells (iPSCs) are cell types with embryonic stem cell (ESC)‐like pluripotency obtained by reprogramming adult cells with the introduction of a variety of exogenous transcriptional regulators [9]. In 2007, researchers used viral transduction technology to successfully transfer OCT4, SOX2, Nanog, and transcription factors such as Lin28 or OCT 3/4, SOX2, KLF4, and c‐Myc into human fibroblasts, obtaining human‐derived iPSCs for the first time [10]. Like ESCs, iPSCs can self‐renew and differentiate into various types of cells and tissues found in the adult body, including the production of blood cells. This makes them a sound model system for hematologic diseases. Meanwhile, iPSCs circumvent some ethical issues and reduce the risk of immune rejection, thereby attracting considerable attention in medical applications.

Currently, the study of α‐Thalassemia is still in the preliminary exploration stage. Especially for TI, the correlation between genotype and phenotype has yet to be further elucidated. Although studies have successfully derived iPSCs from amniotic fluid (AF) cells of healthy or severely affected (Hb Bart’s disease) α‐thalassemia fetuses [11, 12], patient‐specific iPSC models remain lacking for clinically highly heterogeneous intermediate forms of α‐thalassemia, such as the ‐‐^3.7^/α^SEA^ and ‐‐^4.2^/α^SEA^ genotypes. In this study, we established for the first time iPSC lines from fetal AF cells carrying these specific intermediate genotypes. These cell lines will provide a novel in vitro platform for in‐depth investigation of genotype‐phenotype correlations, pathophysiological alterations (particularly in erythropoiesis), and the exploration of new therapeutic strategies for intermediate α‐thalassemia.

## 2. Materials and Methods

### 2.1. Cell Culture and Reprogramming

The study was approved by the Research Ethics Committee of the First Affiliated Hospital of Hainan Medical University (Ethics Approval Number 2023‐KYL‐264). We used an AF cell‐specific medium (Fujifilm) for the expansion culture of AF cells. 1.2 × 10^6^ cells were electroporated with 6 μg of plasmid pEP4‐E02S‐ET2K and 4 μg of pCEP4‐miR‐302‐367 by 4D‐Nucleofector X Unit. The pEP4‐E02S‐ET2K vector expresses OCT4, SOX2, KLF4, and MYC, while the pCEP4‐miR‐302‐367 vector enhances reprogramming efficiency via microRNA cluster expression [13, 14]. After transformation, the cells were cultured in an AF cell‐specific medium. When the confluence reached 50%, the medium was replaced with mTeSR1 (STEMCELL Technologies, 85850). After approximately 10 days of induction culture, we could observe the emergence of morphogenetic clones, and the formed iPSCs exhibited typical ESC morphology. Once the clones grew enough to fill the field of view of a 100x microscope, we picked up and maintained them in mTeSR1 medium on plates coated with Matrigel (Corning, 354277). The medium was changed daily during the culture process, and the cells were dissociated using 0.5 mM EDTA for cell passaging.

### 2.2. Alkaline Phosphatase (AP) Staining

AP staining was performed when the cell density reached 50%–60%, prior to clone attachment. Cells were fixed in 4% paraformaldehyde for 2 min at room temperature. AP buffer was added and equilibrated for 5 min. During equilibration, a freshly prepared AP chromogenic solution is required. We used an AP detection kit (ZOMANBIO: ZD315‐1) containing a set of AP buffer, NBT, and BCIP. For a 35‐mm Petri dish, 1 mL of color development solution was added, prepared in the ratio of AP buffer:NBT: BCIP = 1000 μL:6.6 μL:3.3 μL. After equilibrating, 1 mL of color development solution was added to the Petri dish, and the color development process was carried out for approximately 30 min at 37°C. At the end of color development, the solution was removed and washed twice with PBS. Select the field of view and cell clusters with good staining to be photographed and preserved.

### 2.3. Immunocytochemistry

The cells were fixed in 4% paraformaldehyde for 30 min at room temperature, then washed twice in PBS buffer. Next, cell permeabilization and sealing treatments were performed using a permeabilization solution of 0.2% Triton X‐100 and a sealing solution containing 0.5% BSA for 30 min at room temperature. Then, the cells were treated with primary antibodies overnight at 4°C, followed by secondary antibodies for 1 h at room temperature. The nuclei were counterstained with DAPI for 10 min. Antibodies are listed in Table S1. Images were captured on the confocal microscope (Olympus FV3000).

### 2.4. Quantitative Reverse Transcription Real‐Time PCR (RT‐qPCR)

Total RNA was extracted from iPSCs using Trizol (Invitrogen). cDNA was subsequently synthesized from 1 μg of RNA using the Reverse Transcriptase Kit (Tiangen, Beijing, China). RT‐qPCR was performed by the SYBR Premix Ex Taq TM II kit (cat. no. RR820A; Takara Bio, Inc.) and the Agilent Mx3000P system (Mxpro‐Mx3000P; Agilent Technologies, Inc.). Primers were listed in Table S1. To accurately calculate the relative expression level of mRNA, we applied the 2^−ΔΔCt^ method and selected GAPDH as the normalization control. To ensure the accuracy and reliability of the experimental results, we chose the hESC line (H1) as a positive control (PC).

### 2.5. Karyotype

The cells were treated with 0.14 μg/mL colchicine for 3 h. After completing the co‐culture, the cells were collected and lysed using a 0.075 M hypotonic KCl solution. The cells were then fixed using Carnoy’s fixative (a methanol‐to‐acetic acid volume ratio of 3:1). The cells were transferred to the cytogenetic laboratory for spreading and G‐band cytogenetic analysis by specialized technicians.

### 2.6. Copy Number Variation (CNV) and Thalassemia Mutation Analysis

We collected more than 1 × 10^6^ cells and washed them with PBS buffer to ensure the purity of the cell sample. Immediately thereafter, we forwarded these processed cell samples to the cytogenetic laboratory, where experienced and specialized technicians performed exhaustive CNV analysis and thalassemia mutation analysis on the iPSCs. CNV analysis was performed using array‐based comparative genomic hybridization (aCGH) with a resolution of approximately 100–200 kb, following the manufacturer’s protocol (Agilent Technologies, SurePrint G3 Human CGH Microarray, 8 × 60K). For thalassemia genotyping, genomic DNA was extracted and subjected to multiplex gap‐PCR and PCR‐reverse hybridization assays to detect common α‐globin gene deletions (‐‐^SEA^, ‐α^3.7^, ‐α^4.2^) and mutations.

### 2.7. Teratoma Formation

The 3 × 10^6^ iPSCs were suspended in 30% Matrigel with DMEM/F12 (1:2) and injected into the NOD‐SCID mice subcutaneously (preferably 5–6 weeks old, *n* = 3/group). Significant protrusions were observed at the injection site approximately 50 days after the mice were reared, and surgical methods were used to remove the teratomas. Most of these teratomas showed substantial, but some also exhibited vacuolated features. Teratomas were embedded in paraffin and stained with hematoxylin and eosin (HE). Microscopic analysis examined typical cells within the innermost, intermediary, and outermost germ layers.

### 2.8. Hematopoietic Differentiation of Human Pluripotent Stem Cells (hPSCs)

Before induction, hPSCs were dissociated into single cells using Accutase (Sigma–Aldrich) when they reached 80%–90% confluence. The cells were then seeded onto growth factor‐reduced (GFR) Matrigel (Corning)‐coated six‐well plates at a density of 2 × 10^5^ cells per well. To promote cell survival, the culture medium was supplemented with 0.1 μM Thiazovivin/Y (Selleck Chemicals) for the first 24 h. This day of seeding was designated as Day −1.

The following day (Day 0), the medium was replaced with a specially formulated basal medium (BM) to initiate stepwise hematopoietic differentiation. The BM was composed of DMEM/F12 (Gibco) supplemented with 1% Penicillin–Streptomycin (HyClone), 1% ITS‐G supplement (Gibco), 1% chemically defined lipid concentrate (Gibco), and 70 μg/mL vitamin C (L‐ascorbic acid 2‐phosphate trisodium salt, Sigma–Aldrich). The osmotic pressure of the BM was adjusted to approximately 360–370 mOsm/kg using a 9% NaCl solution prepared in DMEM/F12.

The differentiation protocol was carried out by adding specific cytokines and small molecules to the BM over the subsequent days, with the medium being changed daily [15]. All differentiation procedures were conducted at 37°C under a controlled atmosphere of 5% CO2. The specific cytokine/small molecule combinations for each stage were as follows: Day 0–1:40 ng/mL BMP4 (PeproTech), 30 ng/mL Activin A (Sino Biological), 20 ng/mL bFGF (Sino Biological), 6 μM CHIR99021 (Selleck Chemicals), and 10 μM LY294002 (Selleck Chemicals); Day 1–2: 30 ng/mL BMP4, 1 μM A‐83−01 (Selleck Chemicals), and 2 μM IWR‐1‐endo (Selleck Chemicals). Day 2–4: 40 ng/mL VEGF (Sino Biological) and 50 ng/mL bFGF. Day 4–6: 40 ng/mL VEGF, 50 ng/mL bFGF, 10 μM SB431542 (Selleck Chemicals), 10 ng/mL SCF (PeproTech), 50 ng/mL TPO (Sino Biological), 10 ng/mL IL‐3 (Sino Biological), 50 ng/mL IL‐6 (Sino Biological), and 50 ng/mL FLT3 Ligand (Sino Biological).

### 2.9. Flow Cytometry Analysis

For flow cytometric assessment of pluripotency, undifferentiated iPSCs and the H1 human ESC line (used as a PC) were harvested 3 days after passaging. Single‐cell suspensions were prepared and resuspended in FACS buffer. Surface pluripotency markers were directly stained for 30 min at 4°C in the dark using the following fluorescently conjugated antibodies: PerCP‐Cy5.5 Mouse Anti‐Human SSEA‐4 (clone MC813−70, BD Pharmingen, 561,565), FITC Mouse anti‐Human TRA‐1–81 (BD Pharmingen, 560,883), and FITC Mouse anti‐Human TRA‐1–60 (BD Pharmingen, 560,876). Appropriate unstained cells and isotype controls were included. After staining, cells were washed and resuspended in FACS buffer. Flow cytometric analysis was performed using a BD FACSAria II instrument, and data were analyzed with FlowJo v10.8 software.

To analyze hematopoietic progenitors, cells were harvested and collected by centrifugation at 250 g for 5 min. The resulting cell pellet was resuspended in 100 µL of FACS buffer (DPBS supplemented with 2% fetal bovine serum [FBS]). The cell suspension was then incubated with fluorescently conjugated antibodies against CD34 and CD43 (dilution 1:100) for 30 min at 4°C in the dark. After staining, these cells were washed with FACS buffer, centrifuged, and resuspended with moderate FACS buffer, followed by flow cytometric analysis (BD FACSAria II sorter (BD Biosciences, USA). Data were analyzed using FlowJo v10.8 (FlowJo, LLC).

### 2.10. Colony‐Forming Unit (CFU) Assay

The CFU assay was performed to assess the clonogenic potential of hematopoietic progenitor cells. 5 × 10^3^ HSPCs were resuspended in IMDM medium supplemented with 2% FBS. The cell suspension was thoroughly mixed with 1 mL of methylcellulose medium (MethoCult H4434, Stem Cell Technologies) in 35 mm low‐adherent dishes at 37°C in a humidified atmosphere of 5% CO_2_ for 14–16 days. Colonies derived from erythroid (CFU‐E, BFU‐E), granulocyte‐macrophage (CFU‐GM, CFU‐G, and CFU‐M), and multipotent progenitor cells (CFU‐GEMM) were enumerated based on morphological criteria.

## 3. Results

### 3.1. Reprogramming TI AF Cells

**Figure 1:**
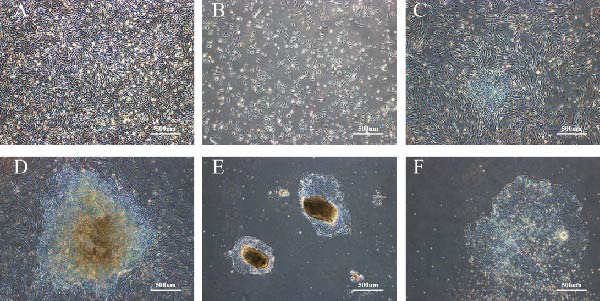
Cell morphology during iPS‐AF2143 induction. (A–D) Cell morphology during induction of iPSCs by AF cells at D0, D3, D14, and D20, respectively. (E) A representative colony of the established iPS‐AF2143 line at passage 1, showing typical pluripotent stem cell morphology (large nucleus, high nuclear‐to‐cytoplasmic ratio, and compact colony). (F) Newly formed colonies after passaging the established iPSCs using EDTA.

The AF cells used in this study were collected from the AF samples of mid‐pregnancy fetuses with prenatal diagnosis of TI. The Ethics Committee of the First Affiliated Hospital of Hainan Medical University approved the study protocol. Taking AF2143 (Figure 1A) as an example, two plasmids, pEP4‐E02S‐ET2K and pCEP4‐miR‐302‐367, were introduced into AF cells by electro‐transfection. On D3, cells resulted to a confluency of approximately 50% (Figure 1B). After 14 days of induction, the cells showed significant clone‐like changes (Figure 1C). During the next 20 days, the cells were cultured and began to exhibit morphological characteristics similar to ESCs—round nuclei, regular arrangement of the cells, and smooth edge of the colonies (Figure 1D). These ESC‐like cells were then isolated, purified, and sub‐cultured for further investigation. On Matrigel‐coated culture plates, the established iPSC lines (exemplified by iPS‐AF2143) stably proliferated and formed typical colonies with smooth edges and prominent nuclei (Figure 1E). Following passaging via EDTA digestion, the cells efficiently re‐formed intact new colonies (Figure 1F).

### 3.2. Karyotype and Thalassemia Detection of iPS‐AF2143

**Figure 2:**
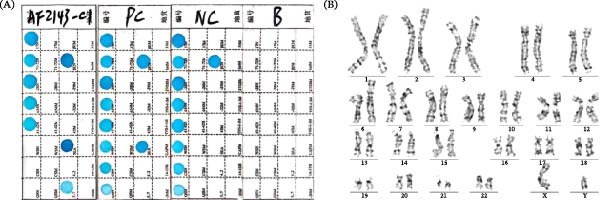
Sequence analysis (A) and karyotype analysis (B) of iPS‐AF2143. (A) Genotyping for α‐thalassemia by multiplex gap‐PCR. Lane PC (positive control): genomic DNA with a known SEA deletion. Lane NC (negative control): wild‐type genomic DNA. Lane B: blank (no‐template control). Lane iPS‐AF2143: sample from the established iPSC line, showing the characteristic band pattern confirming the ‐‐^SEA^/α^3.7^ deletion genotype. (B) G‐banded karyotype analysis. The iPS‐AF2143 line exhibits a normal male karyotype (46, XY) without gross chromosomal abnormalities at the resolution of approximately 5 Mb.

We performed genomic sequence analysis on the iPSCs to ensure that the presence of thalassemia was not altered post‐electroporation. The results confirmed a deletion of ‐α^3.7^/‐‐^SEA^ (Figure 2A), which is consistent with the original fetal amniocentesis diagnosis. In addition, G‐banded chromosome analysis confirmed that the iPS‐AF2143 line possessed a normal 46, XY male karyotype, with no identifiable large‐scale chromosomal changes at this resolution (Figure 2B). However, assessing the genomic integrity of iPSCs requires higher‐resolution detection, as standard G‐banding analysis is insufficient for detecting sub‐microscopic aberrations, which are typically smaller than 5 Mb [16]. Genomic instability, particularly the generation of de novo CNVs, is a significant concern during the reprogramming process. To minimize this risk, we employed a nonintegrating episomal vector method, which has been reported to cause significantly fewer genomic aberrations and CNVs compared to integrating viral methods [17].

Genomic sequence analysis was performed using multiplex gap‐PCR, with PC (DNA with a known SEA deletion), negative control (NC: wild‐type genomic DNA), and B (Blank: no‐template control) included for assay validation. A comprehensive review of all six established iPSC lines confirmed that four lines (iPS‐AF2143, iPS‐AF2444, iPS‐AF2520, and iPS‐AF2392) were free of detectable CNVs. The remaining two lines exhibited minor anomalies (Table 1). iPS‐AF2611 showed a 1.42 Mb deletion at 5q13.2 (clinical significance unclear), and iPS‐AF2219 presented with low‐proportion chimerism for the X chromosome. These findings confirm the relative genomic safety of our nonintegrating reprogramming strategy. Yet, they also underscore the absolute necessity of high‐resolution genomic screening—beyond standard karyotyping—for all iPSC lines to ensure their integrity.

**Table 1:** The CNV results of six Hb H cell lines.

Cytoplasm	CNV
iPS‐AF2143	Negatives
iPS‐AF2611	seq [GRCh37] del(5)(q13.2q13.2) chr5:g.68900001_70320000del 1.42 Mb (clinical significance unclear)
iPS‐AF2444	Negatives
iPS‐AF2219	seq [GRCh37] (X) × 3 [0.19] 155.20 Mb (low proportion chimerism)
iPS‐AF2520	Negatives
iPS‐AF2392	Negatives

### 3.3. Expression Levels of Pluripotency‐Related Markers in iPS‐AF2143

**Figure 3:**
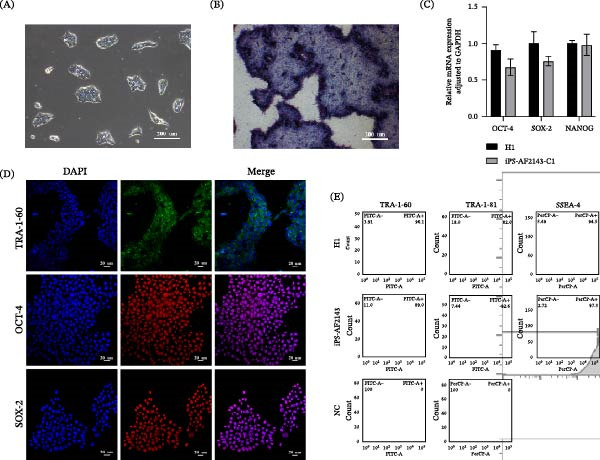
Expression of the pluripotent marker in AF‐iPSC. (A) Bright field and (B) AP staining of iPS‐AF2143. (C) The expression of pluripotent marker OCT‐4, SOX‐2, and NANOG in iPS‐AF2143 by RT‐qPCR. (D) The expression of pluripotent marker TRA‐1‐60, OCT‐4, SOX‐2 in IPS‐AF by immunofluorescence staining. DAPI stained the nucleus (blue). (E) Flow cytometric analysis of pluripotency markers. Histograms showing the expression of TRA‐1‐60, TRA‐1‐81, and SSEA‐4 in the iPS‐AF2143 line compared to the H1.

**Figure 4:**
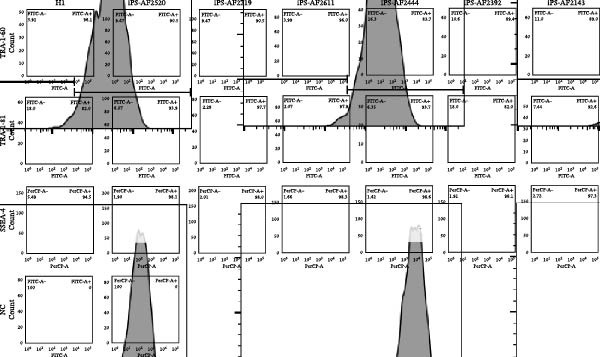
Flow cytometric analysis of pluripotency markers. Histograms display the expression of TRA‐1‐60,TRA‐1‐81, and SSEA‐4 across the established iPSC lines. Each row consists of six panels. The first panel (leftmost) in each row shows the result for the H1 human embryonic stem cell line (positive control). The subsequent five panels (columns 2–6) present the data for the five iPSC lines (iPS‐AF2392, iPS‐AF2444, iPS‐AF2520, iPS‐AF2611, and iPS‐AF2219). The final panel (rightmost) represents the unstained negative control (NC).

We performed a thorough characterization of the iPSCs to demonstrate pluripotency. First, by AP staining experiments, we observed that the cells exhibited a specific blue‐violet color (Figure 3A,B). Second, immunofluorescence (IF) analysis showed that iPSCs expressed typical pluripotency markers: transcription factors OCT‐4 and SOX‐2, and surface antigen TRA‐1–60 (Figure 3D). Real‐time PCR further supported the successful activation of endogenous pluripotency genes OCT‐4, SOX‐2, and NANOG, with expression levels detected and comparable to the PC hESC line (H1) (Figure 3C). While some line‐to‐line variation in expression levels was observed, as is common in qPCR assays comparing different pluripotent cell lines, the robust detection of these core pluripotency transcripts confirms the reprogrammed state. This conclusion is strongly supported at the protein level by the positive IF staining for OCT‐4 and SOX‐2 (Figure 3D). Moreover, flow cytometry analysis was then performed to examine markers mentioned above. The experimental iPSC group exhibited expression profiles consistent with the H1 PC. (Figure 4)

### 3.4. Evaluation of the Differentiation Ability of iPS‐AF2143

**Figure 5:**
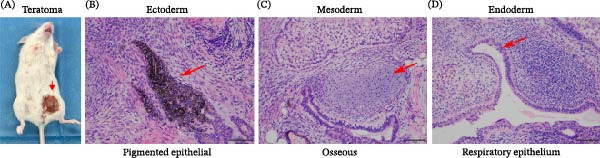
In vivo teratoma formation assay of iPS‐AF2143. (A) Photograph of a surgically resected teratoma from an immunodeficient mouse 6 weeks after subcutaneous injection of iPS‐AF2143 cells. (B–D) H&E staining of teratoma sections, demonstrating differentiation into tissues representative of the three embryonic germ layers. Red arrows point to specific structures: ectoderm (pigmented epithelial), mesoderm (cartilage), and endoderm (glandular epithelium). Scale bars: 100 μm.

**Figure 6:**
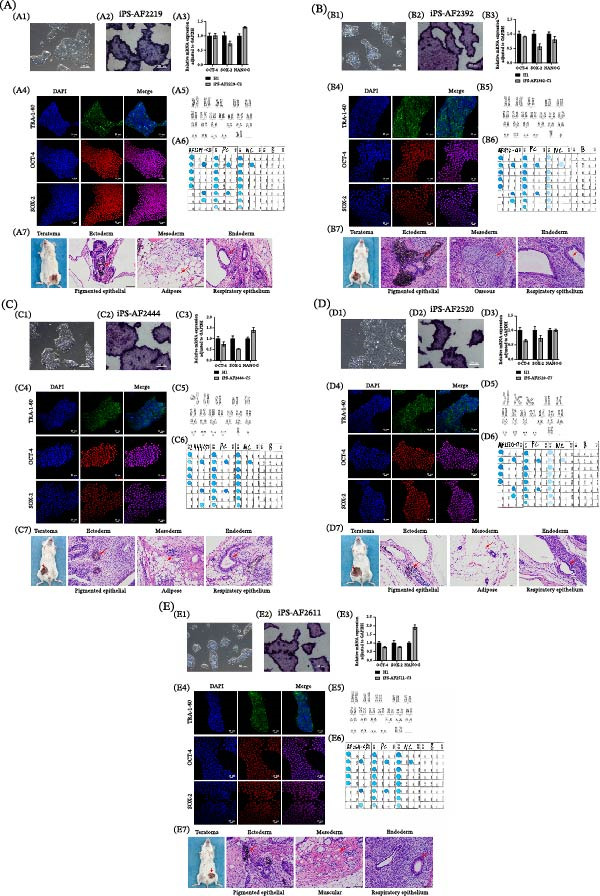
Characterization of the other five established iPSC lines from intermediate α‐thalassemia. It presents key pluripotency validation data for five additional lines (iPS‐AF2392, iPS‐AF2444, iPS‐AF2520, iPS‐AF2611, and iPS‐AF2219). For each line, the following panels are shown: (A1–E1) phase‐contrast image of a typical colony. (A2–E2) AP staining showing positive (purple) activity. (A3–E3) RT‐qPCR analysis for the expression of pluripotency genes OCT‐4, SOX‐2, and NANOG. (A4–E4) Immunofluorescence staining for pluripotency markers SOX‐2, OCT‐4, and TRA‐1‐60; nuclei are counterstained with DAPI (blue). (A5–E5) G‐banded karyotype showing a normal 46, XY or 46, XX karyotype. (A6–E6) Genotyping analysis confirming the retention of the original intermediate α‐thalassemia genotype (‐‐^SEA^/‐α^3.7^ or ‐‐^SEA^/‐α^4.2^). (A7–E7) In vivo teratoma assay. Left panel: Photograph of a resected teratoma. Right panels: H&E staining of teratoma sections demonstrating differentiation into tissues representative of all three germ layers (endoderm, mesoderm, and ectoderm). Scale bars are provided in the respective panels.

Furthermore, we assessed the differentiation ability of the iPSCs by subcutaneously injecting the cells into immunodeficient mice. The morphological analysis revealed that teratoma has three germ layers (Figure 5). Representative tissues from each germ layer are indicated: ectoderm (e.g., neuroepithelial and pigmented epithelial tissue), mesoderm (e.g., osseous, muscular, or adipose tissue), and endoderm (e.g., respiratory epithelium tissue). The other five cell lines were all established using the same method. The results of the identification are shown in Figure 6.

### 3.5. Differentiation Potential of iPS‐AF2520 Into the Hematopoietic Lineage

**Figure 7:**
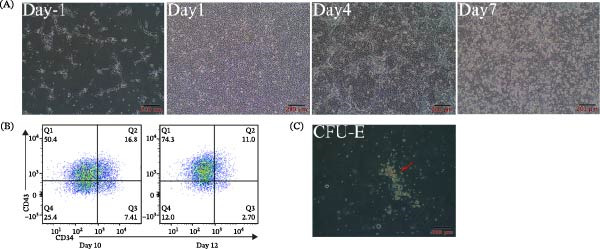
Hematopoietic differentiation of iPS‐AF2520. (A) Morphological changes during in vitro hematopoietic induction. Images show cells on Day −1 (D−1, single‐cell morphology post‐seeding), Day 1 (D1, displaying an endothelial‐like morphology), and Days 4 and 7 (D4 and D7 showing increased cell density and the emergence of hematopoietic endothelial ring‐like structures). (B) Flow cytometry analysis for hematopoietic progenitor markers CD34 and CD43 on Day 10 (D10) and Day 12 (D12). The percentage of CD34^+^/CD43^+^ double‐positive cells is shown in quadrant Q2 (16.8% on D10 and decreasing to 11.0% on D12). (C) Representative images from the colony‐forming unit (CFU) assay performed on D10 progenitors, showing typical hematopoietic colonies and confirming functional hematopoietic differentiation potential.

To evaluate the capacity of the established iPSC lines (using iPS‐AF2520 as an example) to differentiate into hematopoietic cells, we conducted an in vitro directed differentiation experiment. During the early stages of the protocol, distinct morphological changes were observed (Figure 7A). On D−1, the cells exhibited the typical morphology of single iPSCs post‐digestion and seeding. By Day 1 (D1), the cells had become confluent and displayed an endothelial‐like morphology, characterized by smooth edges and tight junctions. In the subsequent induction stages (Day 4 and Day 7), cell density increased, and the morphology gradually became irregular, with the emergence of hematopoietic‐endothelial (HE) ring‐like structures, consistent with differentiation towards the hematopoietic lineage.

We next employed flow cytometry to assess the generation of hematopoietic progenitors by detecting CD34 and CD43 expression on Days 10 (D10) and 12 (D12) of differentiation (Figure 7B). On D10, we detected a CD34^+^/CD43^+^ double‐positive population, accounting for 16.8% of the total cells. By D12, the proportion of this CD34^+^/CD43^+^ population decreased to 11.0%. These results indicate that the differentiation protocol successfully induced the generation of CD34^+^ and CD43^+^ hematopoietic progenitors from iPS‐AF2520.

To further validate the functional clonal potential of these induced progenitors, particularly their erythroid potential, we performed a CFU assay on D10 cells (Figure 7C). The results showed that the cultured cells were capable of forming typical hematopoietic colonies (CFUs), confirming that the iPSC‐derived cells possessed erythroid differentiation potential.

## 4. Discussion

The novelty of this study lies in the first establishment and characterization of iPSC lines derived from fetuses with intermediate α‐thalassemia (‐‐^3.7^/α^SEA^ and ‐‐^4.2^/α^SEA^ genotypes), the construction of cellular model resources specifically for this clinical subtype is unprecedented. However, it is worth noting that the AF samples used in this study were derived from fetuses with specific genotypes of TI, which may limit the generalizability of the results to all patients with TI to some extent. At the same time, differences in genetic backgrounds and disease severity among patients may affect the characteristics of the established stem cell lines.

Regarding genetic diagnosis, most studies have relied only on karyotype analysis to assess the genetic stability of iPSCs. However, karyotype analysis cannot detect chromosomal aberrations smaller than 5 Mb in scale. This would require CNV analysis to detect these. The genomic instability of iPSCs has been a subject of intense discussion, which limits their clinical application [18, 19]. Most studies have relied only on karyotype analysis to assess the genetic stability of iPSCs. In this study, all constructed iPSCs were karyotyped and shown to be expected.

Nevertheless, the CNV analysis of two cases of iPSCs suggested the presence of a small percentage of missing or chimeric genetic material. Generally, copy number changes in a specific gene region directly affect the expression level of that gene. Especially when these changes involve essential developmental or regulatory genes, they may interfere with the normal physiological functions of iPSCs. Considering that iPSCs can differentiate into a wide range of cell types, abnormalities in gene copy number may adversely affect the differentiation potential of iPSCs by disrupting the gene network closely related to cell differentiation, and consequently, the differentiation potential of iPSCs. In the pluripotency validation experiments performed on iPSCs for this study, no significant differences were found between the CNV‐abnormal and normal groups. Despite the deletion or chimerism of genetic material, these iPSCs maintain their pluripotency and differentiation potential.

Thalassemia is a hereditary disease; therefore, research on gene editing and repair using iPSCs has a broad prospect. With CRISPR‐Cas9 gene editing technology, we can correct disease‐causing genes in cell lines, providing a basis for future gene therapy [20, 21]. By differentiating these iPSCs derived from intermediate thalassemia into hematopoietic stem/progenitor cells and ultimately mature erythrocytes, we can model key pathological processes of the disease in vitro, such as ineffective erythropoiesis and abnormal hemoglobin expression [22, 23]. While the present study validated their differentiation into hematopoietic progenitors (Figure 4), the full disease phenotype, such as aberrant hemoglobin expression and ineffective erythropoiesis, manifests during terminal erythroid differentiation. The definitive analysis of the erythrocyte phenotype remains a crucial next step for elucidating the pathological mechanisms of intermediate thalassemia. The established, genotypically well‐defined iPSC lines are essential tools and valuable resources for achieving this goal.

Furthermore, patient‐specific iPSCs allow for testing the effects of potential therapeutic agents on disease‐relevant cells, providing a more precise model for novel drug development [24]. At the same time, this model can also be used to screen existing drugs for potential new uses or side effects, thereby broadening the scope of drug applications [25, 26].

While this study has achieved specific results, many limitations and challenges remain. These cell lines will play a crucial role in the research and treatment of TI. They help us further explore the specific pathogenesis of TI and provide valuable resources and platforms for developing future gene therapies.

**Nomenclature**
CNV: Copy number variation
Hb: Hemoglobin
Hb H: Hb H disease
hESCs: Human embryonic stem cells
HE: Hematoxylin and eosin
iPSCs: Induced pluripotent stem cells
TI: Thalassemia intermedia.

## Author Contributions

Yanlin Ma, Fei Sun, and Yuanhua Huang designed and conducted the study and made the project administration. Fei Sun and Qingyun Chen drafted the manuscript and participated in the data collection. Yanlin Ma and Yuanhua Huang revised the manuscript. Qingyun Chen, Xinxing Jiang, Xinhuai Xue, and Yao Zhou performed data analysis and interpretation.

## Funding

This work was supported by the Major Science and Technology Program of Hainan Province (Grant ZDKJ2021037), the Hainan Province Science and Technology Special Fund (Grants ZDYF2020117, ZDYF2022SHFZ312, and ZDYF2024SHFZ143), the Natural Science Foundation of Hainan Province (Grant 823RC579), project supported by the Hainan Province Clinical Medical Center, and the specific research fund of the Innovation Platform for Academicians of Hainan Province (Grant YSPTZX202310).

## Disclosure

All authors have read and approved the final manuscript.

## Ethics Statement

The study was approved by the Research Ethics Committee of the First Affiliated Hospital of Hainan Medical University (Ethics Approval Number 2023‐KYL‐264).

## Consent

Informed consent was obtained from the participant or all subjects and/or their legal guardians to publish their study as research.

## Conflicts of Interest

The authors declare no conflicts of interest.

## Supporting Information

Additional supporting information can be found online in the Supporting Information section.

## Supporting information


**Supporting Information** Table S1. Antibodies and primers were used in this study. The antibody information for validating iPSC pluripotency and the primer sequences for detecting the expression of pluripotency genes.

## Data Availability

The data supporting this study’s findings are available from the corresponding author upon reasonable request.
