# Supplementary material for: Derivation and Pluripotency Validation of Six iPSC Lines From Amniotic Fluid Carrying Intermediate α‐Thalassemia Genotypes (‐‐3.7/αSEA and ‐‐4.2/αSEA)
Source: Stem Cells Int. 2026 Jun 3;2026:9951684. doi: 10.1155/sci/9951684 (PMC13240418; doi:10.1155/sci/9951684)
Supplement: Supplementary file 1 — Supporting Information Table S1. Antibodies and primers were used in this study. The antibody information for validating iPSC pluripotency and the primer sequences for detecting the expression of pluripotency genes. [file SCI-2026-9951684-s001.docx]

**Supplemental Table 1. Antibodies and primers were used in this study.**

| Antibodies used for immunocytochemistry/flow-cytometry | | | |
| --- | --- | --- | --- |
| Description | Antibody | Dilution | Company Cat # and RRID |
| Pluripotency Markers | Rabbit anti-OCT4 | 1:200 | Cell Signaling Technology Cat# 2750, RRID:AB_823583 |
|  | Rabbit anti-SOX2 | 1:200 | Cell Signaling Technology Cat# 3579, RRID:AB_2195767 |
|  | Mouse anti-TRA-1-60 | 1:200 | Cell Signaling Technology Cat# 4746, RRID:AB_2119059 |
| Secondary antibodies | Goat anti-Rabbit IgG H&L (Alexa Fluor® 594) | 1:1000 | Abcam Cat# ab150080, RRID:AB_2650602 |
|  | Goat Anti-Mouse IgM MU chain (Alexa Fluor@488) | 1:1000 | Abcam Cat# ab150121, RRID: AB_2801490 |
|  |  |  |  |
| Primers |  |  |  |
|  | Target | Forward/Reverse primer (5′-3′) | |
|  | SOX2 | CCCAGCAGACTTCACATGT/  CCTCCCATTTCCCTCGTTTT | |
|  | OCT4 | CCTCACTTCACTGCACTGTA/  CAGGTTTTCTTTCCCTAGCT | |
|  | NANOG | TGAACCTCAGCTACAAACAG/  TGGTGGTAGGAAGAGTAAAG | |
|  | GAPDH | CGAGATCCCTCCAAAATCAA/  TGTGGTCATGAGTCCTTCCA | |
